# Congenital Toxoplasmosis in Chronically Infected and Subsequently Challenged Ewes

**DOI:** 10.1371/journal.pone.0165124

**Published:** 2016-10-27

**Authors:** Thaís Rabelo dos Santos, Gabriela da Silva Magalhães Faria, Bruna Martins Guerreiro, Nathalia Helena Pereira da Silva dal Pietro, Welber Daniel Zanetti Lopes, Helenara Machado da Silva, João Luis Garcia, Maria Cecília Rui Luvizotto, Katia Denise Saraiva Bresciani, Alvimar José da Costa

**Affiliations:** 1 Department of Veterinary Medicine, Rondônia Federal University (UNIR), Rolim de Moura, Av. Norte-Sul, 7300 – Rolim de Moura, RO, 76940-000, Brazil; 2 Department of Animal Pathology, CPPAR, FCAV, UNESP – Jaboticabal, Access road, Prof. Paul Donato Castellane s/n – Jaboticabal, SP, 14884-900, Brazil; 3 Department of Preventive Veterinary Medicine, UEL – Londrina, SP, Brazil; 4 Department of Clinic, Surgery and Animal Reproduction, FMVA, UNESP – Araçatuba, SP, Brazil; 5 Department of Production and Animal Health, FMVA, UNESP – Araçatuba, SP, Brazil; University of British Columbia, CANADA

## Abstract

This experiment studied congenital transmission in sheep experimentally infected with oocysts of *Toxoplasma gondii* and reinfected at one of three stages of pregnancy. Twenty ewes were experimentally infected with *T*. *gondii* strain ME49 (day 0). After the *T*. *gondii* infection became chronic (IFAT≤512), the ewes were allocated with rams for coverage. After the diagnosis of pregnancy, these ewes were allocated into four experimental groups (n = 5): I-reinfected with *T*. *gondii* on the 40^th^ day of gestation (DG); II-reinfected on DG 80; III-reinfected on DG 120; and IV-saline solution on DG 120 (not reinfected). Five ewes (IFAT<64) were kept as negative controls (uninfected, group V), therefore in groups I-III were infected prior to pregnancy and re-infected during pregnancy, group IV was only infected prior to pregnancy, and group V was not infected. Parasitism by *T*. *gondii* was investigated (histopathology, immunohistochemistry, mouse bioassay and PCR) in mothers and lambs tissue. All ewes produced lambs serologically positive for *T*. *gondii*. The results of the mouse bioassay, immunohistochemistry and PCR assays revealed the presence of *T*. *gondii* in all 20 sheep and their lambs. The congenital transmission of *T*. *gondii* was associated with fetal loss and abnormalities in persistently infected sheep and in ewes infected and subsequently reinfected by this protozoan. Therefore, congenital *T*. *gondii* infection was common when ewes were chronically infected prior to pregnancy, with or without reinfection during at various stages of gestation.

## Introduction

Until recently, it was believed that most sheep acquire *Toxoplasma gondii* infection after birth. However, accurate data are not available, and it is thought that < 2% of sheep become congenitally infected with *T*. *gondii* and that < 4% of persistently infected sheep transmit the infection to the next generation[1–3]. These conclusions are based on one recent study [4] and three older studies[5–7]. In Hartley’s study [5] of 38 ewes infected with *T*. *gondii* during a previous pregnancy, all but one ewe gave birth to uninfected lambs, and *T*. *gondii* was isolated from only one placenta [5]. Watson and Beverly studied [7] 26 ewes inoculated with *T*. *gondii* during a previous pregnancy; 24 ewes had uninfected live lambs, one ewe aborted twins, and one ewe was barren. *T*. *gondii* was isolated from the brain of the aborted lamb[7]. Munday [6] studied 178 lambs born to 135 persistently naturally infected ewes; none had pre-colostral *T*. *gondii* antibodies, although the placenta of one ewe was infected with *T*. *gondii*.

Infections acquired early in pregnancy (before 50 days), before the foetus develops the ability to produce antibodies, typically cause embryonic death and reabsorption [5]. If the ewe becomes infected with *T*. *gondii* in the middle of pregnancy (70–90 days), there is a considerable probability of miscarriage or stillbirth[7–9], while in late pregnancy (> 110 days) ewes will give birth normally, although their offspring may be congenitally infected[7, 9]. However, few studies have described the occurrence of newborn lambs that are healthy but infected with *T*. *gondii* in ewe populations[10].

A series of papers was published from a group of researchers[10–15]. These authors proposed that repeat transplacental transmission of *T*. *gondii* in sheep may be more common than previously believed. However, all the evidence they presented was based on the detection of *T*. *gondii* DNA by PCR. These data have also been considered controversial as they go against accepted hypotheses[16]. Edwards and Dubey [17] support the hypothesis that most sheep that have aborted a pregnancy due to *T*. *gondii* develop protection against future toxoplasmosis-induced abortion but that this protection is not absolute.

This investigation aimed to study congenital transmission in ewes experimentally infected and reinfected with *T*. *gondii* oocysts in three gestational stages. We used four laboratory techniques (bioassay, histopathology, immunohistochemistry and PCR) to detect *T*. *gondii* in tissue samples (Central Nervous System, lung, heart, liver, spleen, kidney, skeletal muscle, ovary, uterus and placenta) collected from persistently infected and reinfected ewes and their lambs.

## Materials and Methods

In this study, all procedures using animals complied with the Ethical Principles in Animal Research adopted by the College of Animal Experimentation (COBEA) and were approved (protocol number 024944–08) by the Ethical Committee for Animal Welfare, UNESP, Jaboticabal, São Paulo, (CEBEA).

### Experiment location

The animals were kept isolated in five collection pens in the Sheep Sector of the Research Centre for Animal Health (CPPAR) of the School of Agriculture and Veterinary Sciences (FCAV) of the São Paulo State University (UNESP) in Jaboticabal (21°15’17” S, 48°19’20” W), São Paulo State, Brazil[18].

### Experimental design

The experiment lasted for approximately 12 months. The animals were quarantined for 90 days, and day 0 was defined as the day of the primary infection of the ewes (n = 20). After the *T*. *gondii* infection became chronic (indirect fluorescent antibody test (IFAT) ≤ 512), the ewes were allocated with rams (99 days primary infection) for coverage. After pregnancy confirmation (n = 20), the 25 ewes used in the experiment were divided into five groups of five animals each. Three groups were reinfected (group I: 40^th^ day of gestation (DG); group II: DG 80; and group III: DG 120), one group was primarily infected only (group IV) and one group was uninfected as a negative control (group V). Ewes in groups I-III were infected prior to pregnancy and re-infected during pregnancy, group IV was only infected prior to pregnancy, and group V was not infected.

### Selection of Santa Inês breeding ewes

To select Santa Inês breeding ewes for the experiment, the following physical parameters were examined: heart and respiratory rate, rectal temperature, lymph nodes and overall body condition evaluation, among others. Sonographic examinations were performed to discard pregnant ewes. All the selected ewes were subjected to haematological examination. In the copro-parasitological examination, the nematode eggs per gram of faeces were counted [19] in all ewes during the selection process. All the selected ewes were negative for toxoplasmosis (*T*. *gondii*)[20], neosporosis (*Neospora caninum*)[21], brucellosis (*Brucella abortus*) [22] and leptospirosis (25 serovars: *Andamana*, *Bratislava*, *Australis*, *Butembo*, *Autumnalis*, *Castelollonis*, *Bataviae*, *Canicola*, *Whitcombi*, *Cynopteri*, *Grippotyphosa*, *Sentot*, *Hebdomadis*, *Copenhageni*, *Icterohaemorrhagiae*, *Javanica*, *Panama*, *Pomona*, *Pyrogenes*, *Hardjo*, *Wolffi*, *Patoc*, *Shermani*, *Tarassovi*) [23].

### Selection of Santa Inês breeding rams

Three Santa Inês breeding rams, aged between two and four years, that tested negative for toxoplasmosis, neosporosis, leptospirosis and brucellosis were selected. These males were purchased from the same property as the selected females. Clinical and copro-parasitological exams (oocysts per gram) and complete blood counts were performed in the selection process of these experimental males.

### *T. gondii* strains

#### ME49 strain (primary infection)

Animals were primarily infected using oocysts of the ME49 strain of *T*. *gondii* (type II). ME49 strain used for the ewes infection was kindly provided by Dr. J.L. Garcia (UEL, Paraná, Londrina, Brazil).

#### VEG strain (reinfection)

For reinfection, the primarily infected ewes were inoculated with oocysts of the VEG strain (type III). VEG strain used for the ewes infection was kindly provided by Dr. J.L. Garcia (UEL, Paraná, Londrina, Brazil).

#### RH strain (IFAT antigens)

The slides used in the IFAT were prepared using antigens (tachyzoites) from an RH strain [24] maintained by successive passages in mice in the CPPAR of FCAV/UNESP, Jaboticabal campus.

### Primary infection and reinfection

The primary infection was performed orally using 2.5 x 10^3^ *T*. *gondii* sporulated oocysts of the ME49 strain (type II—non-virulent) for each ewe. Twenty ewes serologically negative for toxoplasmosis and other infectious diseases that could cause fetal loos and abnormalities, such as neosporosis, brucellosis and leptospirosis, were selected for the primary infection. The control group consisted of five females serologically negative (IFAT < 64) for *T*. *gondii* (uninfected).

For reinfection of the 20 primarily ME49-infected ewes, 2.5 x 10^3^ sporulated oocysts of the VEG strain (type III) were used. *T*. *gondii* oocysts of the ME49 (type II) and VEG (type III) strains were administered by a syringe coupled to a metal probe for direct deposition into the animal’s oesophagus. After the inoculation, 100 mL of sterile physiological solution was administered to each animal to clean the syringe and the probe walls, where the oocysts could possibly have adhered (Table 1).

**Table 1:** Experimental design used in the study.

Group	Number of ewes	Title (IFI-IgG) *T*. *gondii* (ME49 strain)	Day of gestation	Days post-primoinfection	Oocysts of the *T*. *gondii* (VEG strain)	Inoculation route
**I**	5	≤512	40	163	2,5 x10^3^	Oral
**II**	5	≤512	80	179	2,5 x10^3^	Oral
**III**	5	≤512	120	219	2,5 x10^3^	Oral
**IV**	5	≤512	-	-	-	-
**V**	5	negative	-	-	-	-

### Oestrus synchronisation programme used in breeding ewes

The oestrus expression in breeding ewes was induced by applying hormones according to the protocol described by Maia[25].

### Clinical examination and laboratory tests

#### Clinical parameters

The physiological parameters evaluated in the ewes were respiratory rate, heart rate and rectal temperature. They were measured in this order, with the animals in the shade, between 8:00 and 10:00 a.m. every two days until the 27^th^ day after the primary infection. As of this date, the exams were performed at seven-day intervals until the end of gestation.

#### Sonographic examination

The experimental ewes were evaluated by ultrasound to confirm the pregnancy. After reinfection, the animals underwent transabdominal ultrasonography every 15 days to assess the evolution of the pregnancy and to detect any changes or foetal losses that might go unnoticed in clinical observations.

#### Immune-humoural response

A search for IgG antibodies against *T*. *gondii* was performed by IFAT in the sera of all ewes, which were obtained from blood samples collected seven days before the primary infection, immediately before the primary infection, every three days until the 30^th^ day after the primary infection and weekly until the end of gestation[20]. Every two weeks, these ewes were subjected to serological tests for brucellosis, leptospirosis and neosporosis. Serology was also performed on foetuses, using pleural fluid or serum, and titres above 32 were considered positive[1]. In lambs born alive and healthy, blood samples were also collected at birth and on the 3^rd^ and 14^th^ days (euthanasia) of life. The three experimental rams were serologically evaluated (brucellosis, leptospirosis, neosporosis and toxoplasmosis) every 15 days.

### Search for *T*. *gondii* in tissue samples (ewe, lambs, stillbirths and/or foetuses)

#### Bioassay in mice

Tissue samples collected from the animals, including from the control group, were inoculated into mice according to the method described by Dubey[26]. The tissues were first cut into small fragments, and connective tissue and fat were removed. Individually, placenta, uterus and ovaries were fully evaluated. For all other organs (spinal cord, brain, lung, heart, liver, spleen, kidney, retina, mammary gland and skeletal muscle and tongue) was performed a pool with all the tissue of the evaluated organs. The tissue pool was homogenised with five volumes of 0.15 M NaCl (saline) using a homogeniser for home use.

Each sample from each animal was inoculated into a group of 15 mice (1 mL/mouse). These mice were observed every day for six weeks [27] for clinical signs of toxoplasmosis. The surviving mice were euthanised [28]to detect antibodies (IFAT) and brain cysts of *T*. *gondii* in serum and brain samples, respectively.

#### Histopathology and immunohistochemistry

For histological examination, the tissues (spinal cord, brain, lung, heart, liver, spleen, kidney, retina, mammary gland, skeletal muscle, ovary, uterus and placenta) were fixed in 10% phosphate-buffered formalin (pH 7.2) for 48 hours and subsequently transferred to a 70% alcohol solution. Then, the material was processed, embedded in histological paraffin, cut into 5-μm pieces and stained with haematoxylin and eosin. Finally, the material was subjected to immunohistochemistry according to the methods detailed by Guesdon[29]. One sample of each tissues (spinal cord, brain, lung, heart, liver, spleen, kidney, retina, mammary gland, skeletal muscle, ovary, uterus and placenta) was evaluated. The histological sections were deparaffinised and hydrated, and the endogenous peroxidase was blocked with a 3% hydrogen peroxide solution. The sections were incubated in a 96°C water bath for 30 min for antigen recovery. The nonspecific binding was blocked by incubating the sections in a solution of milk and 10% bovine serum albumin for 30 min. Subsequently, the sections were incubated for 30 min with primary rabbit anti-*T*.*gondii* antibody (Neomarkers, Fremont, CA, USA) diluted 1:200. Tissue sections were incubated with biotinylated anti-mouse/anti-rabbit antibody (Dako, ADVANCE/HRP kits, US) and then with estrep-tavidin-peroxidase complex (Dako). Afterwards, they were analyzed by avidin-peroxidase, using primary antibody anti-*Toxoplasma* (Neomarker, Fermont, CA, US) with posterior incubation with diaminobenzidin (DAB) developer (Dako), used as the chromogen to reveal the life cycle stages of the parasite, and all samples were counterstained with Harris haematoxylin. Histological sections of sheep brain positive for *T*. *gondii* were used as positive controls for the IHC technique as recommended by the manufacturer, and the primary antibody was omitted for negative controls. The samples were considered positive when bradyzoite pseudocysts were stained in brown by DAB. The animal was considered positive by IHC when at least one of the evaluated organs was positive. The tissue sections were also evaluated in order to search for possible anti-*T*. *gondii* antibody cross reactions with other parasites.

#### *T. gondii* DNA detection by PCR

The collected tissues (spinal cord, brain, lung, heart, liver, spleen, kidney, retina, mammary gland, skeletal muscle, ovary, uterus and placenta) were frozen at -20°C and were subsequently processed according to the technique described by Fuentes [30]. *T*. *gondii* DNA was extracted from the evaluated samples and from the positive control with the DNeasy Blood & Tissues Kit (Qiagen, USA) according to the manufacturer's recommendations. PCR was performed according to the technique described by Fuentes[29]. For detection of *T*. *gondii* DNA in tissue samples, a 194-bp fragment of the B1 gene was amplified using the primers 5’-GGAACTGCATCCGTTCATGAG-3’ (B1_1_) and 5’-TCTTTAAGAGTTCGTGGTC-3’ (B1_2_) as described by Burg [30] and Fuentes [29]. The PCR was performed by adding 500 ng of template DNA to a reaction mix containing 2 mM MgCl_2_, 50 mM KCl, 10 mM Tris-HCl pH 9.0, 0.01% Triton X-100, 0.2 mM dNTPs, 10 pmol of each primer and 5.0 U *Taq* DNA polymerase. The PCR protocol was 2 minutes at 95°C; 35 cycles of 1 minute at 95°C, 30 seconds at 55°C and 1 minute at 72°C; and a final 7 minutes at 72°C. The reactions were performed in a Mastercycler gradient^®^ thermocycler (Eppendorf). The amplified material (15 μL) was analysed by electrophoresis in 2% agarose gel prepared in 1X TAE buffer (Tris-Acetate 40 mM, EDTA 0.1 mM). The electrophoresis was performed in this same buffer at room temperature. Agarose gels containing restriction fragments separated by electrophoresis were stained in an ethidium bromide solution (0.5 μg/mL in water) for 20 minutes and observed with an ultra-violet transilluminator to identify whether the 194-bp fragment, characteristic of *T*. *gondii*, was present.

## Results

### Clinical examination and laboratory tests

#### Clinical parameters

After the primary infection, clinical signs such as hyperthermia, apathy, anorexia and loose stools were observed between days 5 and 7 post-infection. However, after the primarily infected ewes were reinfected (oocysts of the VEG strain—type III), no changes in heart or respiratory rate or rectal temperature that could be attributed to *T*. *gondii* infection were diagnosed.

#### Clinical disorders (reproductive)

The 20 pregnant ewes from groups I, II, III and IV conceived 25 lambs: six from group I, seven from group II, eight from group III and four from group IV. One group was uninfected as a negative control (group V). Fetal loos and abnormalities were registered in the birth period (Table 2)

**Table 2:** Clinical disorders (reproductive) from ewes and their lambs, stillborns or foetuses from groups I, II, III and IV.

Group	Ewe number	Clinical disorders/Number of lambs									
		Healthy lamb	Congenital plantigrade stance in tarsal joints	Arthogryposis with bilateral deviation (varus)	Died two hours after birth	Died three hours after birth	Died four hours after birth	Died 48 hours after birth	Stillborn	Foetus was found in the uterus	Macerated
**GI**	**958**	1	-	-	-	-	-	-	-	-	-
	**970**	-	-	-	-	-	1	-	-	1	-
	**979**	1	-	-	-	-	-	-	-	-	-
	**1039**	1	-	-	-	-	-	-	-	-	-
	**1048**	1	-	-	-	-	-	-	-	-	-
**GII**	**974**	1	-	-	-	-	-	-	-	-	-
	**975**	1	-	-	-	-	-	1	-	-	-
	**980**	2	-	-	-	-	-	-	-	-	-
	**972**	-	1	-	-	-	-	-	-	-	-
	**1016**	-	-	-	-	-	-	-	1	-	-
**GIII**	**1038**	1	-	-	-	-	-	-	-	-	-
	**1019**	-	-	1	-	-	-	-	-	-	-
	**1049**	-	-	1	-	-	-	-	1	-	-
	**1027**	-	-	-	-	1	-	-	-	-	1
	**1041**	-	-	-	-	1	-	-	1	-	-
**GIV**	**1046**	1	-	-	-	-	-	-	-	-	-
	**1023**	1	-	-	-	-	-	-	-	-	-
	**1044**	-	-	-	1	-	-	-	1	-	-
	**1017**	-	-	-	-	-	-	-	-	-	-

#### Sonographic examination

The experimental ewes were evaluated by ultrasound to confirm pregnancy. After reinfection, no changes could be diagnosed in the lambs by ultrasound examination during the entire gestation of all ewes.

#### Immune-humoural response

The seroconversion (IFAT ≥ 64) started five days after the primary infection, and on the 11^th^ day after the primary infection all animals from groups I, II, III and IV showed titres ≥ 64, demonstrating the infectivity of the inoculum used. Ewes maintained as negative controls (G5) remained serologically negative for *T*. *gondii* infection throughout the whole experimental period.

Between the 13^th^ and the 79^th^ day after the primary infection, the maximum serological titres detected were approximately 4,096. After the 93^rd^ day, the maximum titres obtained were 512 until the experimental reinfection. Approximately 20 days after reinoculation with 2.5 x 10^3^ oocysts of the VEG strain of *T*. *gondii*, maximum titres of 2,048 were detected five ewes being in one ewe from group I, one ewe from group II and three ewes from group III.

All ewes remained serologically negative for brucellosis, leptospirosis and neosporosis for the duration of the experimental period.

Table 3 shows that of the 30 lambs born to females from groups I, II, III and IV, 24 lambs had antibody titres against *T*. *gondii* at birth. This initial blood collection was performed before the lambs ingested colostrum. Therefore, the contact these animals had with *T*. *gondii* occurred during pregnancy. In the 14^th^ day of life of the lambs (n = 9) was antibodies remained present and they were euthanised for further detection of *T*. *gondii* through other techniques.

**Table 3:** Antibody titre (IgG) obtained by IFAT and detection of *Toxoplasma gondii* in lambs, stillborns or foetuses from primarily infected ewes (ME49 strain) that were reinfected with 2.5 x 10^3^ oocysts (VEG strain) of *T*. *gondii* and ewes only primarily infected (ME49 strain).

Group	Ewe number/		*T*. *gondii* antibody titre (IFAT)/Days after birth			Detection of *T*. *gondii* (methods)
	Respective lamb		Immediately after birth (pre-colostral)	3 days	14 days	
**I: Reinoculation at 40 days of gestation**	958	Lamb	64	64	32	(B, I and P)
	970	Lamb	64	NP	NP	(I and P)
		Foetus	64	NP	NP	(B and I)
	979	Lamb	64	64	32	(I and P)
	1039	Lamb	128	64	32	(I)
	1048	Lamb	64	64	NP	(B, I and P)
**II: Reinoculation at 80 days of gestation**	972	Lamb	256	64	64	(B, I and P)
	974	Lamb	64	64	32	(B, I and P)
	975	Lamb 1	-	64	NP	(B)
		Lamb 2	128	NP	NP	(B, I and P)
	980	Lamb 1	-	64	64	(B, I and P)
		Lamb 2	128	128	32	(B, I and P)
	1016	Stillborn	128	NP	NP	(B, I and P)
**III: Reinoculation at 120 days of gestation**	1019	Lamb	1024	NP	NP	(B, I and P)
	1027	Lamb	512	NP	NP	(B, I and P)
		Foetus	128	NP	NP	(B and I)
	1038	Lamb	1024	512	128	(B, I and P)
	1041	Lamb	128	NP	NP	(B and I)
		Stillborn	512	NP	NP	(B and I)
	1049	Lamb	512	NP	NP	(B, I and P)
		Stillborn	256	NP	NP	(I and P)
**IV: negative control for reinfection**	1017	Foetus 1	-	NP	NP	(B, I and P)
		Foetus 2	64	NP	NP	(B, I and P)
		Foetus 3	32	NP	NP	(B, I and P)
		Foetus 4	32	NP	NP	(B, I and P)
		Foetus 5	-	NP	NP	(B, I and P)
	1044	Lamb 1	64	NP	NP	(B, I and P)
		Stillborn	32	NP	NP	(B, I and P)
	1046	Lamb	-	≥32	≥32	(B, I and P)
	1023	Lamb	-	≥32	≥32	(B)
**V: negative control for primary infection**	903	-	-	-	-	-
	922	-	-	-	-	-
	956	-	-	-	-	-
	944	-	-	-	-	-
	1051	-	-	-	-	-

-: negative (IFAT < 32); B: Bioassay in mice; I: Immunohistochemistry; P: PCR

NP: not performed

### Search for *T*. *gondii* in tissue samples

#### Bioassay in mice

The bioassay made it possible to detect tissue parasitism by *T*. *gondii* (presence of several brain cysts) in mice inoculated with placenta, ovary, uterus or pooled tissue (skeletal and cardiac muscle, brain/cerebellum, spinal cord, retina, liver, spleen, kidney, lung, tongue and mammary gland) from the ewes from groups I, II, III and IV. *T*. *gondii* was also present in mice inoculated with the tissue pool (skeletal and cardiac muscle, brain/cerebellum, spinal cord, retina, liver, spleen, kidney, lung and tongue) from lambs of the respective females. In ewes from group V (negative control), tissue cysts of *T*. *gondii* were not observed, and all IFAT results were negative. All tissues evaluated in ewes and their respective lambs from groups I, II, III and IV were positive based on the IFAT (titre ≥ 64) that were performed in the respective mice.

#### Histopathology and immunohistochemistry

Histological lesions associated with *T*. *gondii* infection were also observed in tissue samples from the sheep. The lesions from affected tissues were classified as “characteristic” lesions, which were characterized by multiple foci of non-suppurative infiltrates with multifocal necrotic areas surrounded by inflammatory (S4 and S5 Figs). *Toxoplasma gondii* was not detected in the tissue using histopathological examinations. Histopathological lesions were observed only in animals positive for immunohistochemistry (Table 4). These changes were not observed in the control negative ewes, suggesting that the changes found were results of *T*. *gondii* infection. Using immunohistochemistry, *T*. *gondii* could be detected in the animals experimentally infected with *T*. *gondii*.

**Table 4:** Immunohistochemistry results from mice inoculated with tissue fragments obtained from ewes and their lambs, stillborns or foetuses from groups I, II, III, IV and V.

Group	Ewe number		Tissue fragments / Immunohistochemistry												
			Placenta	Ovary	Uterus	Mammary gland	Skeletal muscle	Cardiac muscle	CNS	Lung	Retina	Tongue	Kidney	Liver	Spleen
**I: Reinoculation at 40 days of gestation**	958	Ewe	1	1	1	0	0	0	0	0	0	0	0	0	0
		Lamb	-	-	-	-	1	1	1	0	1	1	1	1	1
	970	Ewe	0	0	0	1	0	0	0	0	0	0	0	0	0
		Lamb	-	-	-	-	1	0	1	0	0	0	0	0	0
		Foetus	-	-	-	-	-	-	-	-	-	-	-	-	-
	979	Ewe	1	0	1	1	0	0	1	0	0	0	0	0	0
		Lamb	-	-	-	-	0	1	1	1	0	1	0	1	1
	1039	Ewe	1	1	1	1	0	1	1	0	0	0	0	0	0
		Lamb	-	-	-	-	1	0	1	0	0	0	0	0	0
	1048	Ewe	1	0	0	0	0	0	0	0	0	0	0	0	0
		Lamb	-	-	-	-	0	0	1	0	0	0	0	0	0
	TOTAL		4	2	3	3	3	3	7	1	1	2	1	2	2
**II: Reinoculation at 80 days of gestation**	972	Ewe	0	0	0	0	0	0	0	0	0	0	0	0	0
		Lamb	-	-	-	-	1	1	0	0	0	0	0	0	0
	974	Ewe	1	1	0	0	0	0	0	0	0	0	0	0	0
		Lamb	-	-	-	-	0	1	1	0	0	0	0	0	0
	975	Ewe	0	0	0	0	0	0	0	0	0	0	0	0	0
		Lamb 1	-	-	-	-	0	0	0	0	0	0	0	0	0
		Lamb 2	-	-	-	-	1	0	1	0	0	1	1	1	1
	980	Ewe	1	0	1	1	0	1	0	0	0	1	0	0	0
		Lamb 1	-	-	-	-	0	0	1	0	0	0	0	0	0
		Lamb 2	-	-	-	-	0	1	1	0	0	0	0	0	0
	1016	Ewe	0	0	1	0	0	0	0	0	1	0	0	1	0
		Stillborn	-	-	-	-	0	1	1	0	0	0	0	0	0
	TOTAL		2	1	2	1	2	5	5	0	1	2	1	2	1
**III: Reinoculation at 120 days of gestation**	1019	Ewe	1	1	0	0	1	0	0	0	0	0	0	1	1
		Lamb	-	-	-	-	0	0	1	0	0	0	0	1	0
	1027	Ewe	1	1	1	0	0	0	0	0	0	0	0	0	0
		Lamb	-	-	-	-	0	1	1	0	0	0	0	0	0
		Foetus	-	-	-	-	1	0	1	0	0	0	0	0	0
	1038	Ewe	1	0	0	1	0	0	0	0	1	0	0	0	0
		Lamb	-	-	-	-	0	1	0	0	0	0	1	0	0
	1041	Ewe	0	0	0	0	0	0	1	0	0	0	0	0	0
		Lamb	-	-	-	-	1	0	1	1	0	0	0	0	0
		Stillborn	-	-	-	-	0	0	1	0	0	0	0	0	0
	1049	Ewe	0	1	1	0	0	0	1	0	0	0	0	0	0
		Lamb	-	-	-	-	0	0	0	1	0	0	1	0	0
		Stillborn	-	-	-	-	0	0	1	1	0	0	0	0	0
	TOTAL		3	3	2	1	3	2	8	3	1	0	2	2	1
**IV: negative control for reinfection**	1017	Ewe	1	1	0	0	0	1	1	0	0	0	0	0	0
		Foetus 1	-	-	-	-	1	1	1	1	0	0	0	0	0
		Foetus 2	-	-	-	-	0	1	0	1	1	0	0	0	0
		Foetus 3	-	-	-	-	0	0	1	1	0	0	0	1	0
		Foetus 4	-	-	-	-	1	1	1	1	0	0	0	0	0
		Foetus 5	-	-	-	-	0	0	1	1	0	0	0	0	0
	1044	Ewe	0	1	0	1	0	1	0	1	0	1	1	0	0
		Lamb 1	-	-	-	-	1	0	0	0	1	0	0	0	0
		Stillborn	-	-	-	-	1	0	0	0	0	0	0	0	0
	1046	Ewe	1	0	0	1	1	0	1	0	0	0	0	0	1
		Lamb	-	-	-	-	0	0	1	0	0	0	0	0	0
	1023	Ewe	1	1	1	1	0	0	0	0	0	0	0	0	0
		Lamb	-	-	-	-	0	0	0	0	0	0	0	0	0
	TOTAL		3	3	1	3	5	5	7	6	2	1	1	1	1
**V: negative control for primary infection**	903	Ewe	-	0	0	0	0	0	0	0	0	0	0	0	0
	922	Ewe	-	0	0	0	0	0	0	0	0	0	0	0	0
	956	Ewe	-	0	0	0	0	0	0	0	0	0	0	0	0
	944	Ewe	-	0	0	0	0	0	0	0	0	0	0	0	0
	1051	Ewe	-	0	0	0	0	0	0	0	0	0	0	0	0
	TOTAL		0	0	0	0	0	0	0	0	0	0	0	0	0
TOTAL			12	9	8	8	13	15	27	10	5	5	5	7	5

0: negative

1: positive

-: not performed

#### *T. gondii* DNA detection by PCR

PCR diagnosed the presence of *T*. *gondii* DNA (Table 5**)** in three, one, four, four and zero ewes from groups I, II, III, IV and V, respectively. As for the presence of *T*. *gondii* DNA in lambs, stillborns or foetuses, we observed DNA amplification of the 194-bp *T*. *gondii* marker sequence in four, six, four and eight lambs from ewes of groups I, II, III and IV, respectively (S5 Fig). Based on the data, it can be inferred that the organs most commonly affected by *T*. *gondii* were from the CNS (16), cardiac muscle (11), skeletal muscle (7), ovary (5), mammary gland (5), liver (5), tongue (5), uterus (4), spleen (4), kidney (4), lung (3), placenta (3) and retina (1). *T*. *gondii* was detected in only one sample of colostrum. *T*. *gondii* DNA was present in 18 samples from the tissue pool of each ewe and their lambs. Considering all the studied organs, *T*. *gondii* was most frequent in the CNS (brain and spinal cord) of experimental ewes and their lambs.

**Table 5:** PCR results from ewes and their lambs, stillborns or foetuses from groups I, II, III, IV and V.

Group	Ewe number		Tissue fragments / PCR													Colostro	Pool
			Placenta	Ovary	Uterus	Mammary gland	Skeletal muscle	Cardiac muscle	CNS	Lung	Retina	Tongue	Kidney	Liver	Spleen		
**I: Reinoculation at 40 days of gestation**	958	Ewe	NR	0	1	0	0	0	0	0	0	0	0	0	0	NR	0
		Lamb	-	-	-	-	1	1	1	0	1	1	1	1	1	-	1
	970	Ewe	0	0	0	NR	0	0	0	0	0	0	0	0	0	NR	NR
		Lamb	-	-	-	-	1	0	1	0	0	0	0	0	0	-	1
		Foetus	-	-	-	-	-	-	-	-	-	-	-	-	-	-	0
	979	Ewe	NR	0	1	1	0	0	1	0	0	0	0	0	0	NR	0
		Lamb	-	-	-	-	0	1	1	1	0	1	0	1	1	-	1
	1039	Ewe	NR	1	1	1	0	1	0	0	0	0	0	0	0	0	NR
		Lamb	-	-	-	-	0	0	0	0	0	0	0	0	0	-	NR
	1048	Ewe	NR	0	0	0	0	0	0	0	0	0	0	0	0	0	NR
		Lamb	-	-	-	-	0	0	1	0	0	0	0	0	0	-	NR
	TOTAL		0	1	3	2	2	3	5	1	1	2	1	2	2	0	3
**II: Reinoculation at 80 days ofgestation**	972	Ewe	0	0	0	0	0	0	0	0	0	0	0	0	0	NR	NR
		Lamb	-	-	-	-	0	1	0	0	0	0	0	0	0	-	1
	974	Ewe	NR	0	0	0	0	0	0	0	0	0	0	0	0	NR	NR
		Lamb	-	-	-	-	0	1	1	0	0	0	0	0	0	-	1
	975	Ewe	0	0	0	0	0	0	0	0	0	0	0	0	0	0	0
		Lamb 1	-	-	-	-	0	0	0	0	0	0	0	0	0	-	0
		Lamb 2	-	-	-	-	1	0	1	0	0	1	1	1	1	-	1
	980	Ewe	NR	0	1	1	0	1	0	0	0	1	0	0	0	1	0
		Lamb 1	-	-	-	-	0	0	1	0	0	0	0	0	0	-	0
		Lamb 2	-	-	-	-	0	1	1	0	0	0	0	0	0	-	NR
	1016	Ewe	0	0	NR	0	0	0	0	0	NR	0	0	NR	0	0	NR
		Stillborn	-	-	-	-	NR	NR	NR	NR	NR	NR	NR	NR	NR	NR	1
	TOTAL		0	0	1	1	1	4	4	0	0	2	1	1	1	1	4
**III: Reinoculation at 120days of gestation**	1019	Ewe	1	1	0	0	1	0	0	0	0	0	0	1	1	NR	NR
		Lamb	-	-	-	-	0	0	1	0	0	NR	0	1	0	-	NR
	1027	Ewe	NR	NR	0	0	0	0	0	0	0	NR	0	0	0	0	NR
		Lamb	-	-	-	-	0	1	1	0	0	0	0	0	0	-	1
		Foetus	-	-	-	-	NR	NR	NR	NR	NR	NR	NR	NR	NR	NR	0
	1038	Ewe	NR	0	0	1	0	0	0	0	NR	0	0	0	0	0	NR
		Lamb	-	-	-	-	0	1	0	0	0	0	1	0	0	-	NR
	1041	Ewe	0	0	0	0	0	0	1	0	0	0	0	0	0	NR	NR
		Lamb	-	-	-	-	NR	NR	NR	NR	NR	NR	NR	NR	NR	-	0
		Stillborn	-	-	-	-	NR	NR	NR	NR	NR	NR	NR	NR	NR	-	0
	1049	Ewe	0	1	0	0	0	0	1	0	0	0	0	0	0	NR	1
		Lamb	-	-	-	-	0	0	0	1	NR	NR	1	0	0	NR	0
		Stillborn	-	-	-	-	NR	NR	NR	NR	NR	NR	NR	NR	NR	-	1
	TOTAL		1	2	0	1	1	2	4	1	0	0	2	2	1	0	3
**IV: negative control for reinfection**	1017	Ewe	1	1	0	0	0	1	1	0	0	0	0	0	0	NR	1
		Foetus 1	-	-	-	-	NR	NR	NR	NR	NR	NR	NR	NR	NR	-	1
		Foetus 2	-	-	-	-	NR	NR	NR	NR	NR	NR	NR	NR	NR	-	1
		Foetus 3	-	-	-	-	NR	NR	NR	NR	NR	NR	NR	NR	NR	-	1
		Foetus 4	-	-	-	-	NR	NR	NR	NR	NR	NR	NR	NR	NR	-	1
		Foetus 5	-	-	-	-	NR	NR	NR	NR	NR	NR	NR	NR	NR	-	1
	1044	Ewe	0	1	0	1	0	1	0	1	0	1	0	0	0	NR	1
		Lamb 1	-	-	-	-	1	0	0	0	0	0	0	0	0	-	0
		Stillborn	-	-	-	-	1	0	0	0	0	0	0	0	0	-	0
	1046	Ewe	NR	0	0	NR	1	0	1	0	0	0	0	0	0	NR	NR
		Lamb	-	-	-	-	0	0	1	0	0	0	0	0	0	-	1
	1023	Ewe	1	NR	NR	0	0	0	0	0	0	0	0	0	0	NR	NR
		Lamb	-	-	-	-	0	0	0	0	0	0	0	0	0	-	NR
	TOTAL		2	2	0	1	3	2	3	1	0	1	0	0	0	0	8
**V: negative control for primary infection**	903	Ewe	-	0	0	0	0	0	0	0	0	0	0	0	0	-	NR
	922	Ewe	-	0	0	0	0	0	0	0	0	0	0	0	0	-	NR
	956	Ewe	-	0	0	0	0	0	0	0	0	0	0	0	0	-	NR
	944	Ewe	-	0	0	0	0	0	0	0	0	0	0	0	0	-	NR
	1051	Ewe	-	0	0	0	0	0	0	0	0	0	0	0	0	-	NR
	TOTAL		0	0	0	0	0	0	0	0	0	0	0	0	0	0	0
TOTAL			3	5	4	5	7	11	16	3	1	5	4	5	4	1	18

0: negative

1: positive

NR: not performed

## Discussion

Clinical (heart and respiratory rate, rectal temperature, lymph node evaluation and body condition), haematological, serological, copro-parasitological and ultrasound examination performed in sheep indicated the health status of the animals in the present study. All the sheep remained negative for neosporosis, brucellosis and leptospirosis for the duration of the experimental period (12 months).

Considering this serological threshold for evaluation of the humoural response in sheep, the animals inoculated with *T*. *gondii* oocysts quickly responded to the antigenic stimulus, showing serological titres ≥ 64 from the 5^th^ day after inoculation. On the 11^th^ day after the primary infection, all animals from groups I, II, III and IV showed titres ≥ 64, indicating the infectivity of the inoculum used. This early humoural response in *T*. *gondii* experimental infections was also detected by Moura [31] in pigs, Arantes [32] in dogs, Lopes [33] in sheep, Scarpelli *et al*. [34]in cattle and Lopes [35] in sheep.

The maximum serological titre (4,096) was detected in ewes primarily infected with *T*. *gondii* from the 13^th^ to the 79^th^ day post-partum. After the 93^rd^ day and before the experimental reinfection, the maximum obtained titre was 512. These data are similar to those observed by Lopes[35], who observed steep decreases of serological titres only from the 63^rd^ or the 70^th^ day after inoculation in sheep inoculated with oocysts or tachyzoites, respectively. Approximately 20 days after reinoculation with 2.5 x 10^3^ oocysts of the VEG strain of *T*. *gondii*, maximum titres of 2,048 were detected in one ewe from group I, one from group II and three from group III. Similarly, Bresciani [36] detected maximum titres of 4,096 in two female dogs after six days of reinfection with *T*. *gondii*. From the 30 lambs born to females from groups I, II, III and IV, 24 had antibody titres against *T*. *gondii* immediately diagnosed at birth (pre-colostral). This fact shows that the contact of these animals with *T*. *gondii* occurred during pregnancy. These results are consistent with those of Lopes[35], who observed the presence of anti-*T*. *gondii* antibodies (IFAT-IgG) at birth (before ingestion of colostrum) in five of the eight lambs from ewes naturally infected with *T*. *gondii*.

*Toxoplasma gondii* was not detected in the tissue using histopathological examinations and the histopathological lesions were observed only in animals positive for immunohistochemistry (Table 3). However, the absence of tissue changes in the control group does not discount these findings. Esteban-Redondo[37], Silva and Langoni [38], Garcia [39] and Lopes [40]noted the difficulty of diagnosing this aetiologic agent in histological sections.

*T*. *gondii* was isolated through the bioassay (the presence of several brain cysts containing bradyzoites) in mice inoculated with placenta, ovary, uterus and pooled tissues from the sheep from groups I, II, III and IV and in mice inoculated with pooled tissue of lambs (seropositive) from their respective mothers that were reinfected by *T*. *gondii*. This result demonstrates that during gestation, tachyzoites of this coccidian passed through the placenta.

The results found by Sharma and Gautam[41], Dubey and Sharma [42] and Dubey [43] corroborate those found in the present study. They isolated *T*. *gondii* from sheep organs through a bioassay after 173 days of inoculation with oocysts and tachyzoites.

The PCR technique made it possible to detect DNA from *T*. *gondii* in ewes and lambs born from ewes of groups I, II, III and IV. *T*. *gondii* was detected in only one sample of colostrum. *T*. *gondii* DNA was present in 18 samples from the tissue pool of each ewe and their lambs. Considering all the studied organs, *T*. *gondii* was most frequently detected in the CNS (brain and spinal cord). Similar results were found by Esteban-Redondo and Innes[44], who detected *T*. *gondii* (isolated M3) more frequently in the brain and in the cardiac muscle of experimentally infected ewes.

The lower parasitism in some genomic samples of reinfected sheep (mothers and lambs) that was detected by PCR compared to the bioassay does not imply the absence of *T*. *gondii* from the portion of tissue used for the PCR or some parasites may have been lost in the DNA extraction procedure. Therefore, the "genomic" DNA (host + parasite) in each reaction might have contained a low amount of parasite DNA that was insufficient to visualise the amplification of 194 bp in a 2% electrophoresis gel stained with ethidium bromide[45].

According to Esteban-Redondo and Innes[44], in a study of experimental *T*. *gondii* infection in ewes, the parasite was more consistently detected by PCR in the group of ewes infected with 10^5^ oocysts than in the group infected with 10^3^ oocysts. Therefore, it can be inferred that the lower positivity obtained by PCR in this study compared to the bioassay in mice might have been related to the concentration of the inoculum used (2.5 x 10^3^).

Some authors advocate the combination of PCR-based toxoplasma detection techniques with other diagnostic methods[46, 47]. The mouse bioassay's superiority compared to PCR has also been verified in pig tissues or semen by Garcia[39], Tsutsui [48] and Moura[31, 32], in dogs by Arantes[32], in sheep by Lopes *et al*. [33]and Lopes[35], in cats by Montoya [49] and in cattle by Scarpelli *et al*.[34].

The results from group IV (only primarily infected) support the suggestion of Buxton[2], i.e., the congenital transmission may be more frequent than expected in ewes persistently infected with *T*. *gondii*, most likely due to acute relapse of the infection. Therefore, the hypothesis that primary infection protects against reinfection, justifying the decision by many sheep farmers to not discard ewes with an abortion history, must be rejected. In this study, ewes persistently infected and reinfected did not have abortions; however, severe changes occurred (locomotive changes, malformations, stillbirths and debility) in their lambs. This is consistent with the findings of Morley [13] which showed that breeding from infected ewes presented a high risk of infection and abortion.

In a recent study of a hamster model, congenital transmission of *Toxoplasma* during the chronic stage of infection in the mother has been observed[50]. Other researchers have observed similar results in hamsters [51] and, infrequently, in the rat[52], and it has been studied in the ewe, and other mammals, in nature[12, 14]. Recently, a group of researchers from England [10–13]proposed that repeated *T*. *gondii* transplacental transmission may be more common in sheep than previously believed. However, all the evidence presented was based on *T*. *gondii* DNA detection by PCR[53]. These findings allow us to presume that the hamster model works in a similar way to that in nature, wherein pregnant women and ewes that experienced a toxoplasma infection previously protect their foetuses against infection with the parasite during pregnancy[8, 54]. Only few exceptions to this situation have been reported in women [55] and in ewes[12]. The results found in the present study are consistent with the findings reported by Duncanson[10], Morley[11], Williams[12], Morley[13], Edwards and Dubey[17].

## Conclusions

In summary, ewes persistently infected with *T*. *gondii* transmitted the infection congenitally, possibly due to an acute relapse process. This result shows that the immunity acquired in the primary infection did not protect the ewes against future *T*. *gondii* reinfections. The experimental *T*. *gondii* reinfection triggered severe reproductive alterations (locomotive changes, malformations, stillbirths and disability) in Santa Inês ewes primarily infected at different pregnancy stages. Therefore, congenital *T*. *gondii* infection was common when ewes were chronically infected prior to pregnancy, with or without reinfection during at various stages of gestation.

## Supporting Information

S1 Fig Skeletal dysmorphogenesis were characterised by plantigrade stance in tarsal joints (lamb 972). (TIF)

S2 Fig Arthogryposis with bilateral deviation (varus) in stifle joints (lamb 1019). (TIF)

S3 Fig Arthogryposis with bilateral deviation (varus) in stifle joints (lamb 1049). (TIF)

S4 Fig Focal coagulation necrosis associated with mononuclear infiltrate the myocardium. (TIF)

S5 Fig Non-suppurative infiltrates in the lung interstitium. (TIF)
