# Supplementary figures and images for: Congenital Toxoplasmosis in Chronically Infected and Subsequently Challenged Ewes
Source: PLoS One. 2016 Oct 27;11(10):e0165124. doi: 10.1371/journal.pone.0165124 (PMC5082944; doi:10.1371/journal.pone.0165124)

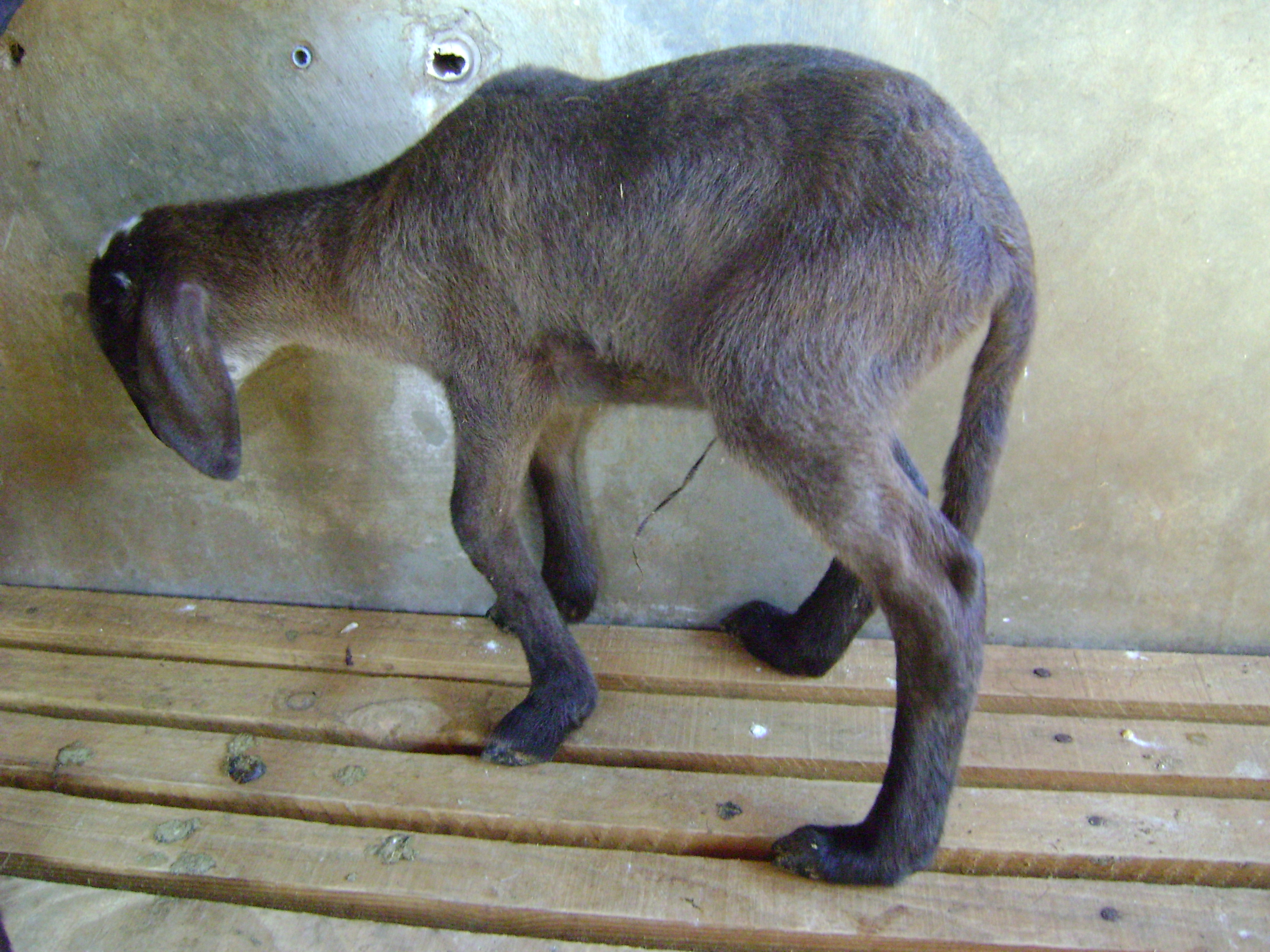

Supplement: S1 Fig — (TIF) [file pone.0165124.s001.tif]

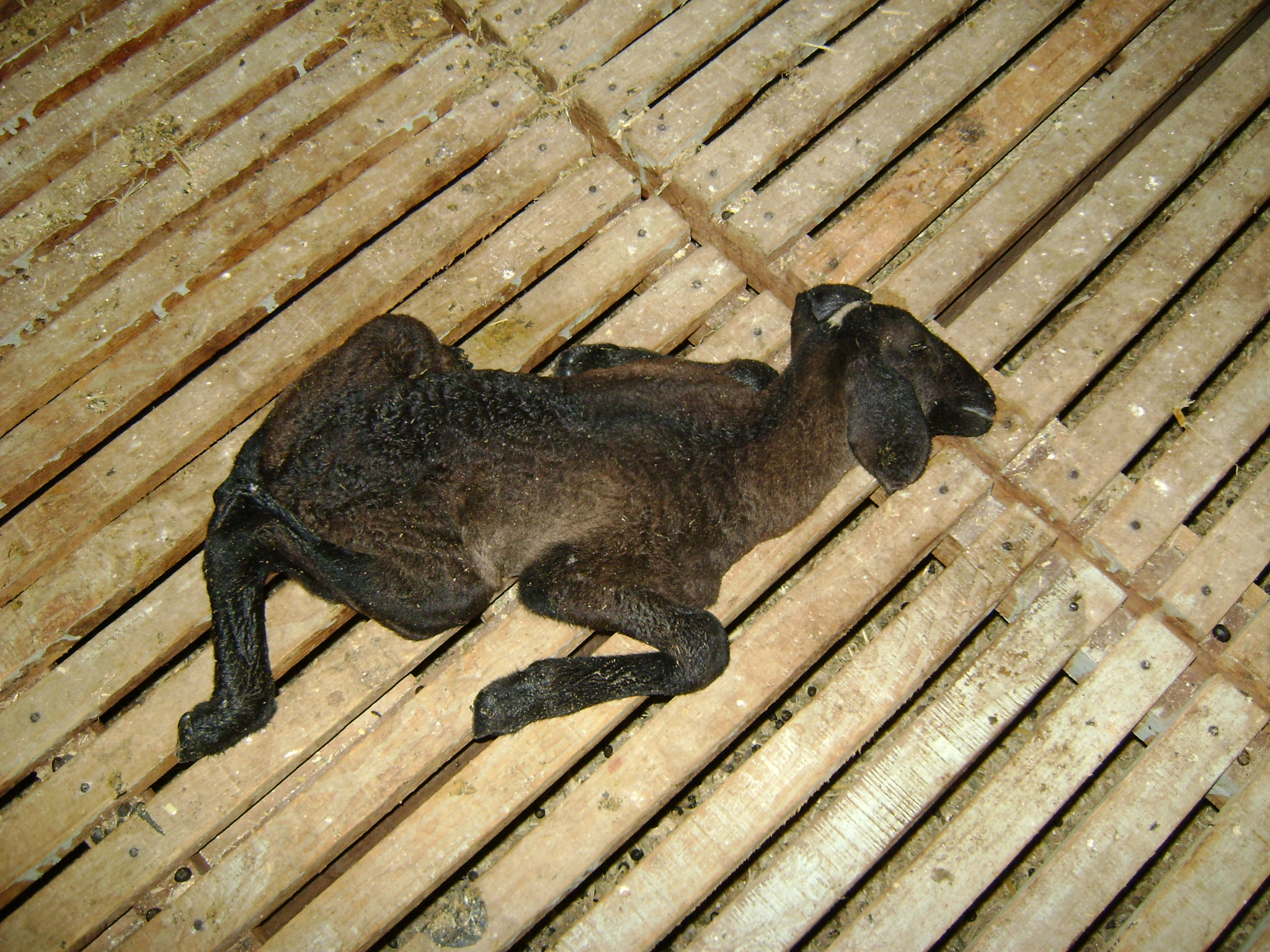

Supplement: S2 Fig — (TIF) [file pone.0165124.s002.tif]

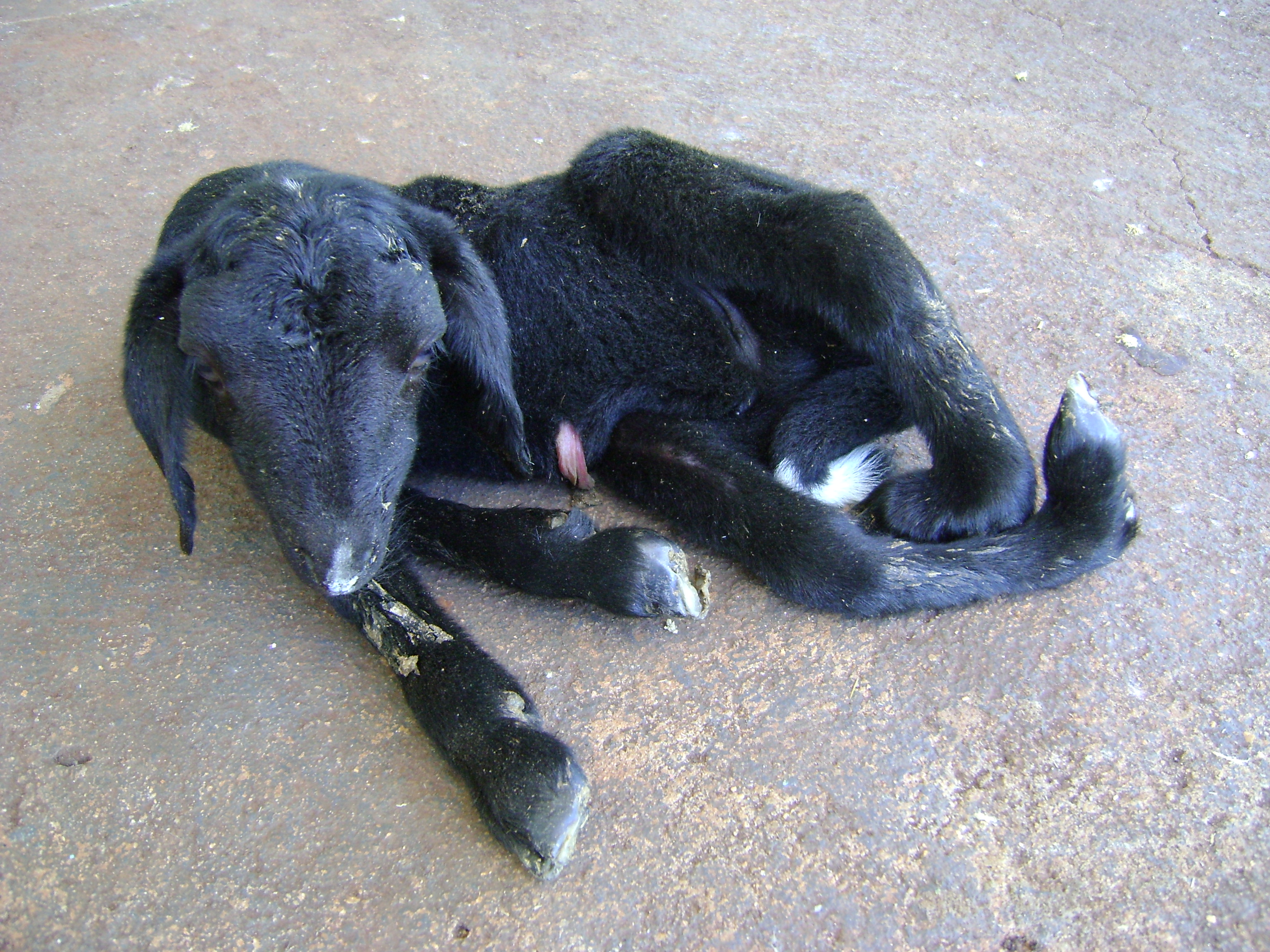

Supplement: S3 Fig — (TIF) [file pone.0165124.s003.tif]

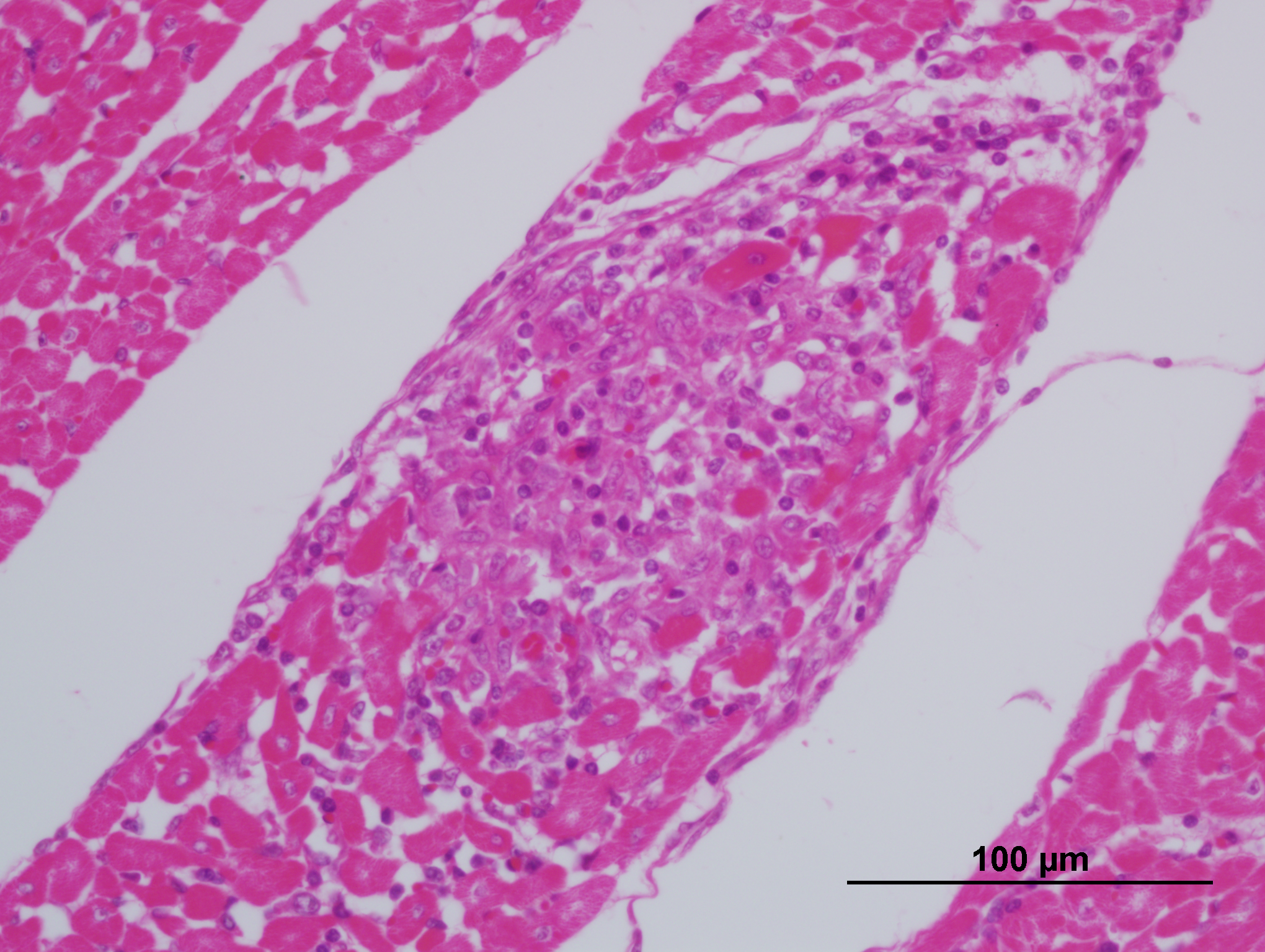

Supplement: S4 Fig — (TIF) [file pone.0165124.s004.tif]

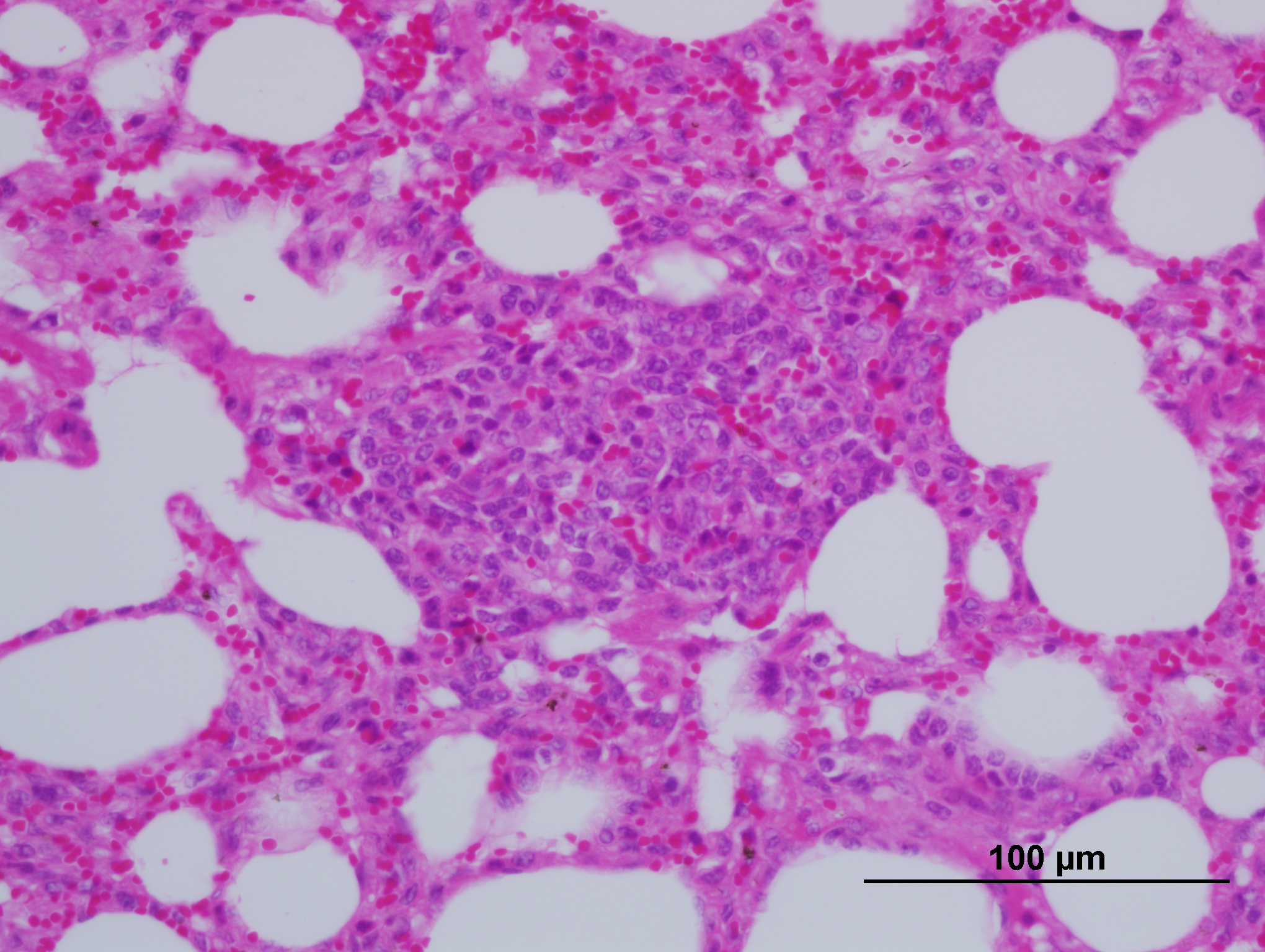

Supplement: S5 Fig — (TIF) [file pone.0165124.s005.tif]
